# Complete Genome Sequences of Two Waterfowl-Origin Tembusu Virus Strains Isolated in Shandong Province, China

**DOI:** 10.1128/genomeA.00789-13

**Published:** 2013-12-19

**Authors:** Hao Chen, Xin Liu, Yi Tang, Ying Zhang, Jinfeng Ti, Xuhui Gao, Youxiang Diao

**Affiliations:** College of Animal Technology and Medicine, Shandong Agricutural University, Tai’an, Shandong Province, Chinaa; Shandong Vocational Animal Science and Veterinary College, Weifang, Shandong Province, Chinab

## Abstract

Here, we report the complete genome sequences of two tembusu virus strains, ZC-1 and LQ-1, isolated from ducks and geese, respectively, in 2012. Phylogenetic analysis showed that the nucleotide and amino acid sequences of the two strains are closely related to those of the TMUV isolates around Shandong province. The full-length genome sequences of two waterfowl-origin TMUVs provided herein will help to understand the molecular epidemiology of tembusu virus in China, which deserves further investigation.

## GENOME ANNOUNCEMENT

The flavivirus tembusu virus (TMUV) was originally isolated from a duck in China in 2010 (1). Since then, outbreaks of the disease have been reported frequently in many provinces in eastern and southern China. The affected ducks manifest the clinical symptom of heavy egg-drop syndrome. The production rates of the infected layer ducks decreased from 90% to 10%, or even less (1, 2). In this study, genomes of two TMUV isolates were sequenced and analyzed.

In October 2012, two TMUV strains were isolated from a duck farm with an outbreak of egg-drop syndrome in Peking ducks and another goose farm with an outbreak of neurological symptoms in wulong geese in Shandong province, China. Samples from the theca folliculi from the affected ducks and the brain from the affected geese were used for virus isolation, and the two isolated virus strains were named ZC-1 and LQ-1. Seven pairs of overlapping PCR primers were designed in our lab using Primer Premier 5.0 software, according to two published complete genome sequences of TMUV, SD001 and the sparrow-isolated strain SDMS (GenBank accession no. KC333867) (2–4). According to the manufacturer’s instructions, RNA extraction was used for the synthesis of viral cDNA using the M-MLV reverse transcriptase (Takara) with seven reverse primers, as designed. Seven overlapping fragments of TMUV cDNA were amplified using *Ex Taq* polymerase. The PCR products were purified and sequenced (Invitrogen). The sequences were assembled and manually edited to produce the full-length genomic sequence of the TMUV isolates.

The full-length genome sequences of the two waterfowl-origin tembusu virus strains are 10,990 nucleotides (nt) in length, contain a long open reading frame (ORF) (95 nt to 10,230 nt), encode a polyprotein of 3,425 amino acids (aa), and contain three structural proteins (capsid, PrM, envelope) and eight nonstructural proteins (NS1, NS2A, NS2B, NS3, NS4A, 2K, NS4B, and NS5). Comparative sequence analysis revealed that ZC-1 and LQ-1 are 98.1% to 99.0% identical to other TMUV strains. The percent identity of the nucleotide sequences of the genomic sequences of these two isolates is 99.7%. The two strains were simultaneously isolated from two farms that are in two adjacent cities. Specifically, the nucleic acid and amino acid sequences of the two strains are more closely related to the strains BYD-1 (5) and JS804 (6), isolated in the provinces around Shandong, and a duck-origin strain isolated in Shandong (4).

Although waterfowls infected with TMUV exhibited heavy egg-drop syndrome or encephalitis as their main symptom, the layer hens and sparrows infected with TMUV did not exhibit any symptoms or pathological changes. Serum samples collected from people who worked in the TMUV-infected farms were tembusu virus positive (7). Whether TMUV poses any risk to people is not currently known.

We report herein the complete genome sequences of two tembusu virus strains, ZC-1 and LQ-1. The data contribute to the exploration of the molecular epidemiology and evolutionary of the tembusu virus. Moreover, the mutation sites of different strains might reveal the mechanisms of molecular epidemiology and molecular pathogenesis.

### Nucleotide sequence accession numbers.

The complete genome sequences of the duck and goose tembusu virus isolates have been submitted to GenBank under the accession no. KF557894 and KF557893, respectively.
